# Protoplast isolation prior to flow cytometry reveals clear patterns of endoreduplication in potato tubers, related species, and some starchy root crops

**DOI:** 10.1186/s13007-017-0177-3

**Published:** 2017-04-14

**Authors:** F. Parker E. Laimbeer, Sarah H. Holt, Melissa Makris, Michael Alan Hardigan, C. Robin Buell, Richard E. Veilleux

**Affiliations:** 1 Department of Horticulture, Virginia Tech, Blacksburg, VA 24061 USA; 2 Department of Biomedical Sciences and Pathobiology, Center for Molecular Medicine and Infectious Diseases, Virginia-Maryland Regional College of Veterinary Medicine, Virginia Tech, Blacksburg, VA 24061 USA; 3 Department of Plant Biology, Michigan State University, East Lansing, MI 48824 USA

**Keywords:** Solanaceae, Endopolyploidization, *Solanum tuberosum*, Karyoplasmic ratio

## Abstract

**Background:**

Endoreduplication, the process of DNA replication in the absence of cell division, is associated with specialized cellular function and increased cell size. Genes controlling endoreduplication in tomato fruit have been shown to affect mature fruit size. An efficient method of estimating endoreduplication is required to study its role in plant organ development. Flow cytometry is often utilized to evaluate endoreduplication, yet some tissues and species, among them the tubers of *Solanum tuberosum*, remain intractable to routine tissue preparation for flow cytometry. We aimed to develop a method through the use of protoplast extraction preceding flow cytometry, specifically for the assessment of endoreduplication in potato tubers.

**Results:**

We present a method for appraising endoreduplication in potato (*Solanum tuberosum*) tuber tissues. We evaluated this method and observed consistent differences between pith and cortex of tubers and between different cultivars, but no apparent relationship with whole tuber size. Furthermore, we were able to observe distinct patterns of endoreduplication in 16 of 20 wild potato relatives, with mean endoreduplication index (EI) ranging from 0.94 to 2.62 endocycles per cell. The protocol was also applied to a panel of starchy root crop species and, while only two of five yielded reliable flow histograms, the two (sweet potato and turnip) exhibited substantially lower EIs than wild and cultivated potato accessions.

**Conclusions:**

The protocol reported herein has proven effective on tubers of a variety of potato cultivars and related species, as well as storage roots of other starchy crops. This method provides an important tool for the study of potato morphology and development while revealing natural variation for endoreduplication which may have agricultural relevance.

**Electronic supplementary material:**

The online version of this article (doi:10.1186/s13007-017-0177-3) contains supplementary material, which is available to authorized users.

## Background

Endoreduplication is the replication of the nuclear genome without subsequent cytokinesis, resulting in cells that have greater DNA content than somatic cells remaining in the mitotic cycle. The exact purpose of this alternate cell cycle process remains enigmatic but it is thought to play a role in differentiation and specialization of cells and is common in plants, especially within storage and nutritive tissues [1–3]. One hypothesis, predicated on the positive correlation between cell size and nuclear DNA content, is the ‘karyoplasmic ratio’; that an increase in nuclear size and DNA content is required to maintain homeostasis with the large cell sizes necessitated by certain storage, secretory, or structural functions [4, 5]. It may be tempting to reduce the role of endoreduplication in cell size determination to one of direct causality; however, the real picture is considerably more complex. For instance, Chevalier et al. [6] demonstrated that manipulation of cell cycle regulatory genes may alter the endoreduplication index (EI), the mean number of replication cycles per cell, and concomitantly the size of the tomato fruit itself. Conversely, experiments in Arabidopsis have shown that ectopic overexpression of EI promoting genes often yields plants with severe dwarfism, perhaps due to perturbations in the normal organizational structure of the developing plant [7]. Additional research is required to understand the nuanced influences endoreduplication has on plant organ size if it is to be a potential target for selective breeding or other approaches in crop improvement.

As Chevalier et al. [6] demonstrated profound effects caused by alterations of EI on tomato fruit, we aimed to investigate the extent of endoreduplication in another nutrient sink tissue, potato tubers. Typically, endoreduplication can be assessed by flow cytometry through the use of crude tissue preparations or fixations and DNA-binding fluorophores [8, 9]. Fluorescent intensity is directly correlated with DNA content, meaning that for each endocycle a given cell has undergone, its fluorescent intensity will double relative to a 2C somatic cell. The relative abundance of peaks corresponding to different nuclear DNA contents (C-values) can be observed and an average number of endocycles per cell, the endoreduplication index, can be calculated [10]. Unfortunately, we found the protocol that we routinely employ to establish ploidy and endoreduplication in potato leaves, which uses a crude preparation in a modified Galbraith’s buffer, yielded poor and unreliable results when applied to tuber preparations, likely due to the abundance of starch-storing amyloplasts and/or modified cell wall composition [11, 12]. To the authors’ knowledge there are only two research articles that describe investigation of tuber endoreduplication levels, both of which paired acetic acid fixation to pectinase digestion [13, 14]; upon attempting this approach, we occasionally obtained reasonable histograms but found the protocol to be unreliable. Previously, protoplast generation prior to flow cytometry has been used to reduce debris and increase clarity of histograms for other plant tissues [15–17]. After digestion of the cell wall and extracellular matrix, free cell suspensions can easily be obtained, potentially allowing for more effective release of nuclei and greater binding of the fluorophore to the DNA while removing the effects of complicating debris. Isolation of potato tuber protoplasts has also been reported [18–20]; however no protocol combining the use of tuber protoplasts with flow cytometry for the evaluation of endoreduplication has been described. The goal of this research was to develop such a protocol, assess the breadth of its application, and employ it to investigate how endoreduplication varies among tuber tissues, tuber sizes, and accessions of potato species.

## Methods

### Plant material

Field grown tubers from the Montcalm Research Farm, Michigan, of *Solanum tuberosum* Group Tuberosum cv. Superior were kindly provided by David Douches (Michigan State University) and used within 8 weeks of harvest to optimize the protocol and then compare the EI between tuber cortex and tuber pith in samples from small (10–20 g), medium (50–60 g), or large (>100 g) tubers. The tissue and tuber size experiment was performed using a randomized complete block design with three replications, blocked by day. A single longitudinal core was taken of each tuber and the innermost 1 cm of tissue was used as the pith sample while the outermost, excluding the epidermis, 5 mm sections from each side of the core were combined for the cortex sample. The protocol was evaluated on a wide range of tuber-bearing *Solanum* spp. comprising a diversity panel, using greenhouse tubers harvested from 3-month old plants grown from February to May in East Lansing, MI under 23 °C days and an increasing 11–14 h ambient photoperiod, then stored at 4 °C/95% RH for 8–9 weeks prior to sampling. Due to variation in storage response, some tubers were in poor condition (dehydrated, soft, or signs of fungal rot) prior to sampling; however that does not appear to have entirely precluded success with the protocol. To accommodate the variation in tuber size and shape, we used an entire longitudinal core for sampling, with some exceptions. Small tubers (<1.5 cm length) were prepared using a scalpel to recreate a longitudinal core. For the largest tubers (>6 cm length), the longitudinal core was halved in the center, as to avoid overtaxing the digestion solution, and the distal half was used. To evaluate the protocol on a range of starchy vegetables, we purchased plant material for a panel of other starchy root and tuber crops from a local grocery store: Beets (*Beta vulgaris* subsp. *vulgaris*), carrots (*Daucus carota* subsp. *sativus*), turnip (*Brassica rapa* subsp*. rapa*), cassava (*Manihot esculenta*), sweet potato (*Ipomoea batatas*), and potatoes of different varieties and pigments (*Solanum tuberosum;* purple, red, yellow, russet). The carrots and beets were marked as organic; otherwise their history and origin are unknown. Here, no blocking was performed and all samples were processed on the same day. A 1 cm central fraction of a longitudinal core of the pith was used for all samples with the exception of cassava (*Manihot esculenta*) where cortex was taken because the pith is tough and inedible. For the ImageStream analysis and protoplast images, cv. Superior was used for both the leaf and tuber pith samples. Leaves were taken from in vitro plants while the tubers were treated as above.

### Tuber flow cytometry protocol

**Fig. 1:**
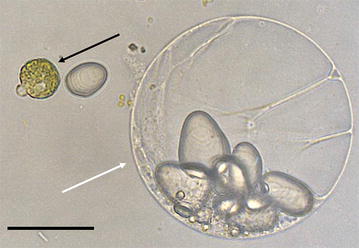
Image of leaf (*black arrow*) and tuber (*white arrow*) protoplasts prior to the addition of modified Galbraith’s flow cytometry buffer. A starch granule lies between the two protoplasts and many more are visible within the tuber protoplast. *Scale bar* = 50 µm

A visual diagram of the protocol is displayed in Additional file 1. The protoplast isolation steps are similar to previously published potato tuber protoplast protocols with some modifications [19, 20]. Washed tubers were surface sterilized by submersion in 75% ethanol for 5 min and then allowed to dry under a laminar flow hood. A longitudinal core was taken from the apical to the basal end using a heat sterilized 7 mm cork borer. Depending on the experiment and thus tissue being sampled, a specific 1 cm section of the core was taken and sliced into approx. 3 mm^3^ pieces and placed into a 50 ml conical centrifuge tube containing 15 ml filter-sterilized (0.45 μm) plasmolysis incubation solution (PIS) (0.55 M mannitol, 2 mM CaCl_2_, 1 mM KH_2_PO_4_, 1 mM MgCl_2_, 50 mM Tris buffer, pH adjusted to 7.5 with HCl) and incubated overnight at 4 °C. Following plasmolysis, the incubation solution was aspirated off with a sterile serological pipette and 10 ml of filter-sterilized (0.45 μm, aPES or other low-protein-binding) enzyme solution [0.71 M mannitol, 3 mM CaCl_2_, 1 mM KH_2_PO_4_, 1 mM MgCl_2_, 4% “Onozuka” R-10 cellulase (Yakult Pharmaceutical Co. Ltd., Tokyo, Japan), 0.8% macerozyme R-10 (Yakult Pharmaceutical Co. Ltd., Tokyo, Japan), 1% hemicellulase (Sigma-Aldrich, St. Louis, MO), 10 mM MES buffer, pH adjusted to 5.8 with NaOH] was added. The samples were then incubated overnight (18–20 h) at 29 °C at 180 rpm horizontal shaking. At this point, some deterioration of the tissue was usually visible but often the pieces remained partially intact. After digestion, aseptic technique was no longer necessary. Next, the samples were gently shaken, then allowed to rest for 5 min to allow the protoplasts to settle. The enzyme solution was removed using a serological pipet and replaced with 15 ml modified plasmolysis incubation solution (PW) (0.71 M mannitol, otherwise identical) and the tubes inverted to wash the protoplasts (Fig. 1). After another 5 min rest, the wash solution was then aspirated off, using a pipette to remove as much residual liquid as possible without disturbing the pellet. Next, 1.5 ml of ice-cold modified Galbraith’s buffer (13.6 mM sodium citrate-trisodium, 8 mM MOPS, 18 mM MgCl_2_, 0.4% v/v Triton X-100) were added to each sample prior to 2–5 s vortexing to break up the aggregated protoplasts. Samples were immediately placed on ice and passed through a 106 μm mesh filter into a 2 ml microcentrifuge tube. Then, RNase A dissolved in modified Galbraith’s buffer was added to each sample to a final concentration of 0.16 mg/ml prior to a brief inversion and a 30 min incubation at RT. Finally, each sample received propidium iodide (PI), also dissolved in modified Galbraith’s solution, to 0.04 mg/ml, the tube was then inverted to mix, and incubated on ice for at least 15 min. Samples were allowed to sit on ice for no longer than 2 h prior to flow cytometry.

### Flow cytometry

The stained nuclei samples were analyzed with a BD FACSCalibur (BD Biosciences, San Jose, CA) flow cytometer. Propidium iodide fluorescence was measured with 488 nm laser and a 585/42 bandpass filter. Data were analyzed with FlowJo VX software (Treestar, Inc, Ashland, OR). Nuclei were gated using a FSC-H vs log-scale PI-H plot to remove debris. At least 2000 gated events were collected per sample. Median fluorescent intensity and frequency were calculated for nuclei populations and reported in arbitrary units (AU). Endoreduplication index (EI) represents the mean number of endocycles per cell and was calculated using the following equation: EI = [*4C*] + 2[*8C*] + 3[*16C*] + 4[*32C*] + 5[*64C*] where [*4C*] is the percentage of 4C nuclei, [*8C*] is the percentage of 8C nuclei and so on.

For the ImageStream, nuclei samples of tubers and in vitro leaves of potato cv. Superior were obtained through the method described above. Samples were analyzed, imaged, and sized with an Amnis ImageStream Mark II (Amnis Corp, Seattle, WA) imaging flow cytometer. PI fluorescence was measured with 488 nm laser and a 610/30 bandpass filter. Data were analyzed with IDEAS software (Amnis Corp, Seattle, WA). Nuclei were gated using an area versus fluorescent intensity plot to visualize populations and remove debris.

## Results

### Tuber flow cytometry protocol

**Fig. 2:**
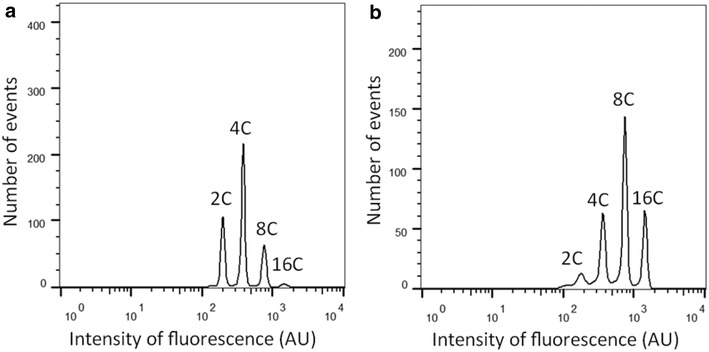
Example of flow cytometry histogram obtained from protoplast preparation of *Solanum tuberosum* cv. Superior tuber cortex (**a**) and pith (**b**) showing relative abundance of nuclei with differing DNA contents. *Note* the separation between peaks allowing for reliable frequency estimations

The development of a reliable and straightforward means to investigate the levels of endoreduplication in potato tubers and other starchy organs was the major goal of this project. The success of the protocol can be evaluated by examining the clarity and separation of peaks in the flow cytometry histograms (Fig. 2). Over the course of developing and validating the protocol we noted a few critical steps which greatly influenced the quality of results. Foremost, as much of the PW solution as possible should be removed in the step following enzyme digestion and preceding the addition of the modified Galbraith’s buffer. As little as 100 μl remaining high osmotic pressure PW solution is enough to severely degrade the nuclei rendering the sample useless. It is not clear if this is due to the osmotic pressure itself or activity of residual enzyme but, during troubleshooting, this step was found to be the most frequent cause of failed samples. Thus, while a serological pipette is appropriate for aspirating during other steps of this protocol, greater care must be taken to ensure complete removal of liquid prior to release of nuclei via the Galbraith’s buffer. The other critical step is the vortexing or vigorous shaking of the sample after the addition of the modified Galbraith’s buffer. Here, much of the tissue will remain aggregated despite the enzyme treatment and it is necessary to break up these aggregates to ensure complete exposure of the protoplasts to the buffer as required for nuclei release. Since different genotypes may vary in degree of digestion, the duration of the vortex or shake will vary by sample; however care must be taken not to ‘over-vortex’ as damage to the nuclei may be incurred. For instance, samples of cv. Superior required only 2–3 s of vortexing while at least 5 s were necessary for PI 234011 samples. Another note of some significance is the use of aseptic technique to ensure samples are not contaminated prior to incubation and digestion. While care was taken to avoid such contamination, it was nevertheless observed in a few samples. From our experience, bacterial contamination did not render a sample entirely useless but rather ‘messier’ with less distinction between peaks of different C-values which was presumably caused by bacterial damage to protoplasts and the nuclei they contain. Hence, it is possible that aseptic technique may not be necessary for certain gross applications requiring maximum throughput but remains best practice for obtaining the highest quality results. Lastly, all samples should be run on the flow cytometer within 2 h of the addition of the modified Galbraith’s buffer as after this window we saw considerable decline in sample quality as well as peak shifting, indicating deterioration of nuclei. It is possible this could be avoided through the use of fixatives however this has not been tested.

### Tissues of cv. Superior

To test the consistency of results obtained with the protocol, as well as examine how EI differs across sizes and tissues of potato tubers, we evaluated the cortex and tuber tissues of various tuber sizes of cv. Superior. We observed a significant difference in endoreduplication levels between the cortex and pith tissue across tuber sizes (p = 0.002); however there was no significant difference due to tuber size itself (p = 0.89). Pith of cv. Superior had an average EI of 1.73 endocycles per cell while cortex tissue yielded an average of 1.31. Next, we applied the protocol to a diverse panel of semi-domesticated and wild *Solanum* species to test if the protocol could accommodate samples with potentially different cell wall composition and variable amounts of cellular and extracellular debris.

### *Solanum* spp. diversity panel

**Fig. 3:**
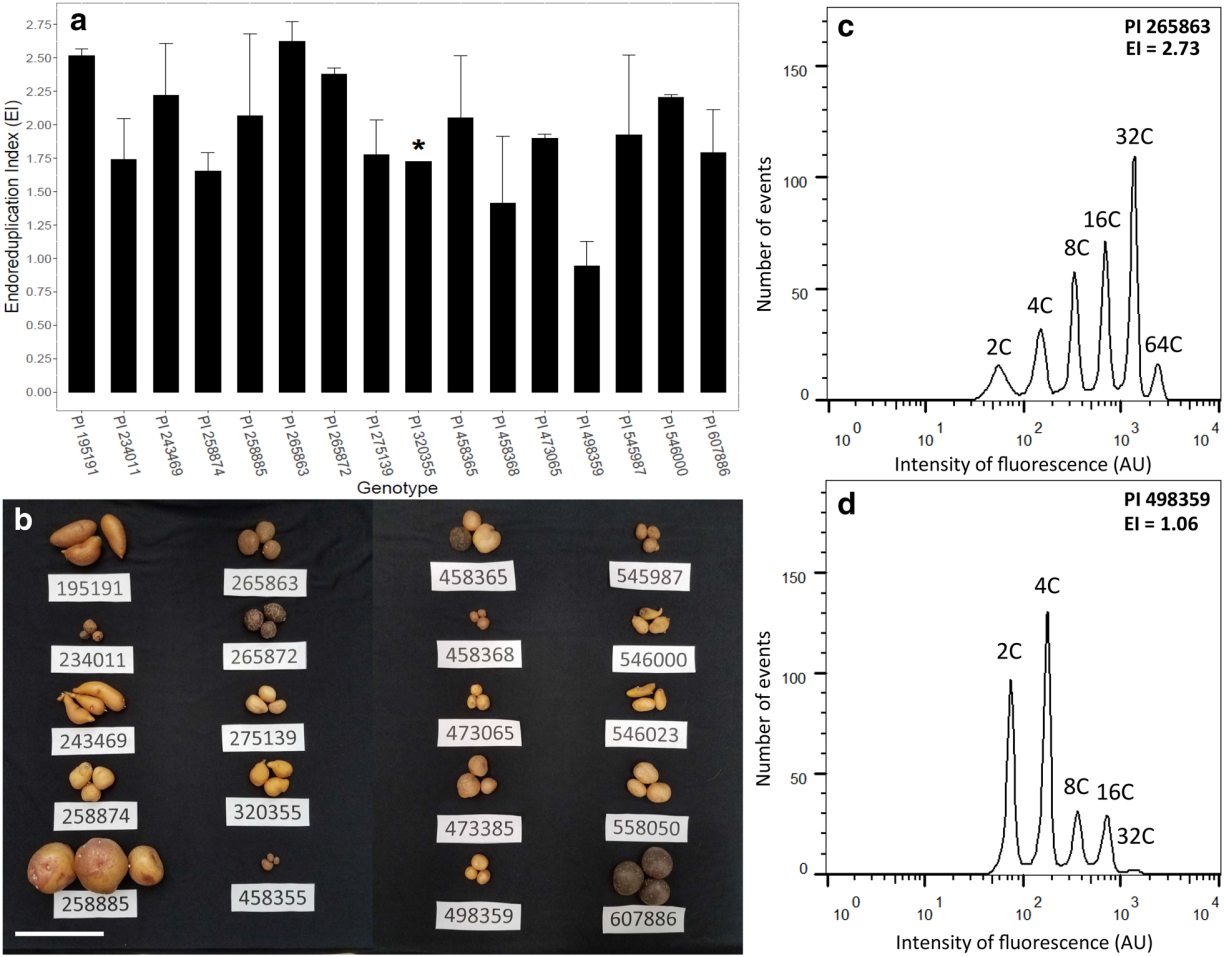
The results of the tuber endoreduplication protocol applied to the diversity panel. **a** Shows mean EI for each accession using a longitudinal core. *Asterisk* indicates genotype with only one successful rep. **b** Displays the tubers at the time of sampling, including those genotypes for which successful results were not obtained. *Note* variation in tuber size, shape, and rigidity. *Scale bar* = 10 cm. **c**, **d** Show representative flow cytometry histograms and EI values of the genotypes with the *highest* (C, PI 265863) and *lowest* (D, PI498359) mean endoreduplication values

We found the protocol was successful on at least one sample for the majority (16/20 tested) of the accessions in the diversity panel indicating its application, while broad, is not universal. Of note is that these genotypes varied greatly in tuber quality after storage and one of the unresponsive genotypes, PI 458355, was of particularly poor quality at the time of sampling (Fig. 3b). Of the other three unresponsive lines, PI 473385 and PI 546023 appeared to have limited or no digestion after the enzyme incubation while PI 558050 seemed to oxidize rapidly, despite being promptly immersed in the plasmolysis solution. We observed significant differences among the responsive genotypes of the diversity panel (Fig. 3a). For instance, PI 498359, a diploid *S. kurtzianum* accession, demonstrated the lowest mean EI of the panel at 0.94 while the greatest was PI 265863, a diploid *S. candolleanum* line, with a mean EI of 2.62 (Fig. 3c, d). It should be noted that the diversity panel exhibited greater sample-to-sample variation than either the cv. Superior tissue samples or other cultivar tubers and potential reasons are considered in the discussion.

### Cultivars and root crop species

**Fig. 4:**
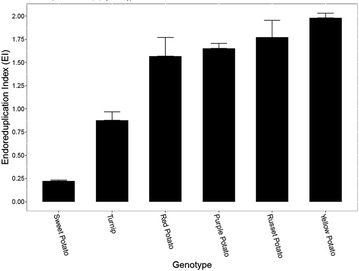
Mean EI values obtained with the protocol for the tuber and tuberous root crop panel. Unresponsive species are not shown

Next, we elected to apply the protocol on a variety of other root crops to further evaluate its effectiveness on other starchy plant organs wherein endoreduplication levels may be germane to development. We included four store-bought potato varieties (purple, red, russet, yellow) with unknown histories of growing conditions, harvest, storage and exact cultivar identities to investigate if significant EI differences exist among cultivated potato varieties. We observed slight but significant differences between some of the potato varieties. For instance, yellow potato averaged 1.98 endocycles per cell while the red variety displayed a mean 1.56 per cell. Of the other starchy root crops, only turnip and sweet potato yielded distinct peaks while cassava, beet, and carrot did not. Due to variation in nuclear DNA content we expected to observe some shifting of the 2C peak compared to potato. As expected, we observed a minor decrease in fluorescent intensity from potato to sweet potato and an even further decrease in turnip. This is in accordance with previous literature where potato has been found to contain 3.32–3.86 pg/2C nucleus whereas sweet potato and turnip contain 3.31 and 1.06 pg, respectively [21]. Nevertheless we observed moderate levels of endoreduplication in turnip, although much lower than what was observed in potato and has been previously reported in fruits of many crop species. Sweet potato demonstrated low levels of endoreduplication with an average of only 0.2 endocycles per cell (Fig. 4). A summary of all evaluated species and accessions is presented in Table 1 and all raw data for successful samples are available in a supplementary file (see Additional file 2).

**Table 1:** Potato accessions and other species used for flow cytometric analysis of starchy tissues

Species	PI number	Ploidy	Response
*S. tuberosum*	195191	2*x*	+
*S. tuberosum*	234011	2*x*	+
*S. tuberosum*	243469	2*x*	+
*S. tuberosum*	258874	4*x*	+
*S. tuberosum*	258885	4*x*	+
*S. candolleanum*	265863	2*x*	+
*S. medians*	265872	2*x*	+
*S. chacoense*	275139	2*x*	+
*S. tuberosum*	320355	2*x*	+
*S. microdontum*	458355	2*x*	−
*S. berthaultii*	458365	2*x*	+
*S. okadae*	458368	2*x*	+
*S. brevicaule*	473065	2*x*	+
*S. brevicaule*	473385	2*x*	−
*S. kurtzianum*	498359	2*x*	+
*S. brevicaule*	545987	2*x*	+
*S. boliviense*	546000	2*x*	+
*S. tuberosum*	546023	4*x*	−
*S. commersonii*	558050	2*x*	−
*S. tuberosum*	607886	4*x*	+
*S. tuberosum* cv. Superior	–	4*x*	+
*S. tuberosum* (russet)	–	4*x*	+
*S. tuberosum* (red)	–	4*x*	+
*S. tuberosum* (yellow)	–	4*x*	+
*S. tuberosum* (purple)	–	4*x*	+
*Brassica rapa* subsp*. rapa (*turnip)	–	2*x*	+
*Manihot esculenta (*cassava)	–	2*x*	−
*Beta vulgaris* subsp. *vulgaris* (beet)	–	2*x*	−
*Daucus carota* subsp. *sativus* (carrot)	–	2*x*	−
*Ipomoea batatas (*sweet potato)	–	6*x*	+
Total	–	–	23/30

*PI* plant introduction

Response: + acceptable flow cytometry histograms obtained; − no meaningful histograms obtained

### ImageStream flow cytometry

**Fig. 5:**
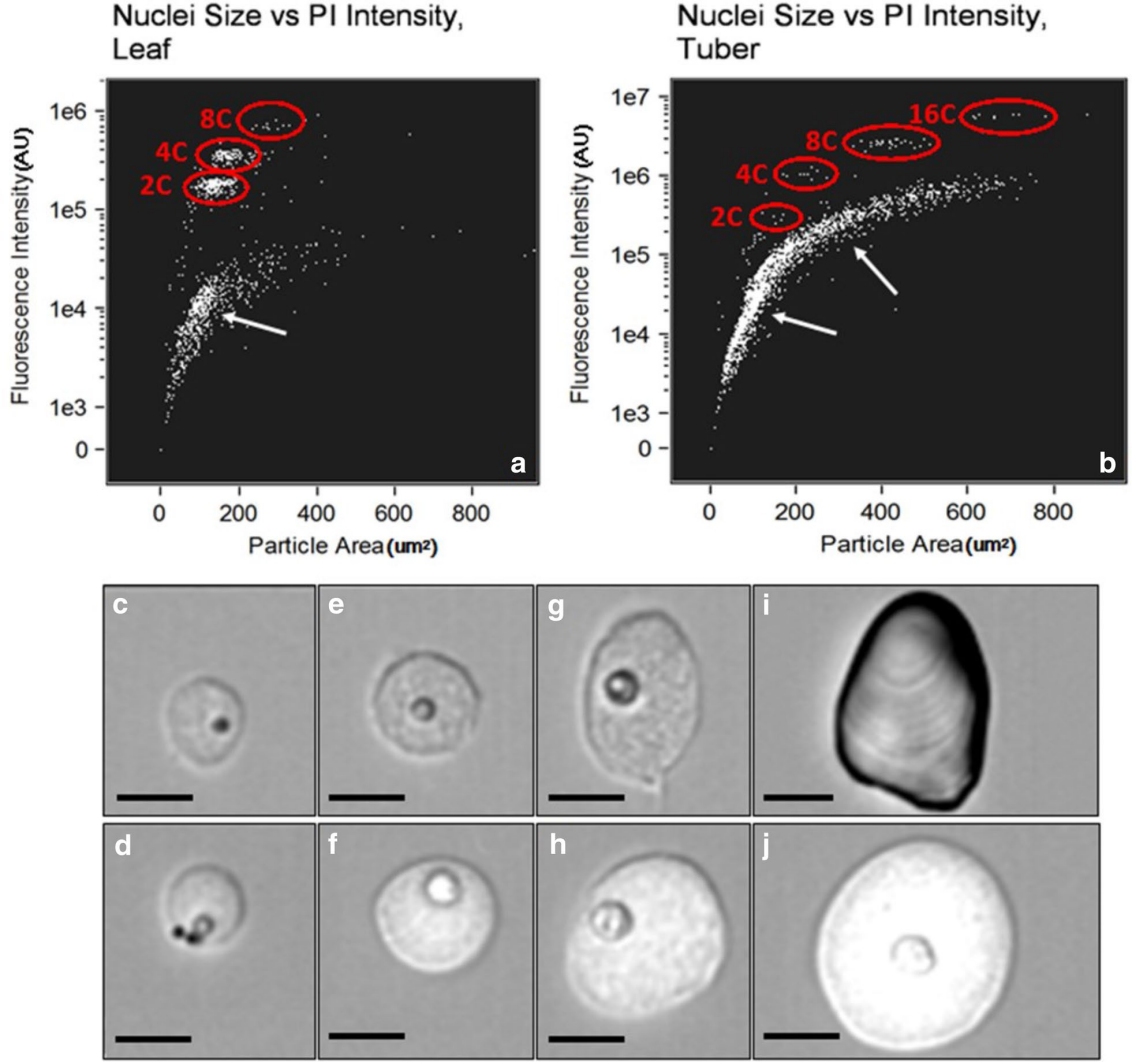
Relationship between fluorescence intensity and particle area in leaf (**a**) and tuber pith (**b**) nuclei obtained from protoplast preparations. Populations of different C-values are *circled*. Other points represent debris (*arrows*) **c**–**j**: bright-field images obtained during ImageStream flow cytometry of leaf (**c**, **e**, **g**) and tuber (**d**, **f**, **h**, **j**) nuclei obtained from protoplast preparations with increasing C-values which are representative of the populations circled in **a**, **b**. **i** Depicts a large starch granule from the “debris” in **b**. *Scale bar* = 10 μm. *Note* scatterplots are meant to be illustrative and were not used to calculate relative abundances of nuclei due to lower flow rate of ImageStream cytometer

To verify that the protocol identified and classified nuclei based of their size and fluorescence intensity we employed an ImageStream flow cytometer to capture images of potato cv. Superior nuclei and other particles which were released during the sample preparation. Hence, the ImageStream allowed us to produce the same results as the FACSCalibur flow cytometer, albeit at a slower pace, with the notable advantage of being able to capture an image of each event. With the ImageStream, we were able to verify the relationship between nucleus size and fluorescent intensity as evidenced by the separate populations observed (Fig. 5a, b). Furthermore, a representative nucleus, one of the events depicted in panels A and B, from each C-value population is displayed to illustrate the increase in size that accompanies each endocycle (Fig. 5c–j). Taken together, this demonstrates the stepwise increase in DNA content in an endopolyploid nucleus is accompanied by an increase in total nucleus size and greater intensity of fluorescence which is then detectable through the protoplast-flow cytometry protocol.

## Discussion

### Tuber flow cytometry protocol

Here, we describe a novel protocol for the evaluation of endoreduplication levels of potato tubers; a phenomenon which has previously received limited investigation despite its relevance to development and potential as a target for crop improvement. We demonstrate that the protocol is effective on an assortment of potato relatives as well as some tuberous root crops. The protocol requires two overnight incubations as well as the process of sterilizing and coring the tuber samples which may be time consuming. Therefore, while it is demonstrably appropriate for small to moderate sized experiments, the throughput may limit its application in ventures requiring high-throughput analysis such as fine mapping or assessment of large quantities of breeding stock. Some protoplast isolation methods, such as that presented by Doke and Tomiyama, utilize a shorter enzymatic incubation period which we found to garner protoplast quantities insufficient for flow cytometry [19]. Apart from the time investment, another limitation of the approach is the high cost of reagents, particularly enzymes. To mitigate this, researchers may consider using a smaller volume of enzyme solution and/or tissue per preparation. We have observed satisfactory results with as much as 3 cm of core (7 mm diameter) in 10 ml of enzyme solution, indicating some tolerance for adjustment in the ratio of enzyme to tissue. Alternatively, it may be possible to reuse enzyme through filtration and subsequent pH adjustment as reported in Saxena and King [22] where similar yields were observed reusing enzyme solution as many as two times; however, this was not attempted here. Finally, researchers aiming to make additional improvements to the protocol may consider altering the type and ratios of the enzymes utilized for protoplast isolation. For instance, the protocol presented herein employs Onozuka R-10 cellulase which may contain impurities, including nucleases, thus potentially damaging nuclei [23, 24]. More purified cellulase preparations are available (e.g., Onozuka RS), albeit at increased cost, which may expand the protocol to recalcitrant accessions or improve the quality of data obtained. While the protocol does have its shortcomings, we believe it represents a substantial step forward for the assessment of endoreduplication in root and tuber crops, as evidenced by the differences we observed between tissues, accessions, and species.

### Tissues of cv. Superior

The results of our experiment on the chipping cultivar, Superior, indicate that, at maturity, there is a difference between the EI of cortical and pith tissues but no apparent relationship to overall tuber size. It has been previously noted from microscopy work that, especially in the latest stages of development, perimedullary parenchyma, in the form of cell division and enlargement, is the predominant contributor to increases in total tuber size, while pith and cortex tissues are comparatively static [25]. Additionally, prior research has demonstrated that across the developmental spectrum, pith cells are consistently larger than cortical cells and may therefore be expected to display greater levels of endoreduplication [26]. Hence, our results are consistent with prior literature as even the smallest tubers we examined (10–20 g) were well beyond the initial stage where cortical and pith division and enlargement are most abundant (<8 mm). Further research must be performed to determine if the increase in cell number and size in the perimedullary tissue during later development is accompanied by increases in EI. Additionally it would be interesting to determine the degree to which maximum attainable tuber size of various cultivars is dictated by increases in cell size versus cell proliferation.

### *Solanum* spp. diversity panel

The evaluation of the *Solanum* species panel establishes that the protocol’s application extends beyond cultivated potato and thus might be appropriate for diversity and domestication studies. The differences observed in these wild and semi-domesticated lines demonstrate substantial natural variation that may have been selected for in the domestication of potato. Furthermore, considering that some of these tubers had stored poorly or displayed defects (dehydration, fungal growth, hollow heart) yet the majority were still responsive shows the protocol may be robust to other tuber quality defects such as tuber end rot. Three of the accessions responded poorly despite minimal visual decline in storage. For two of these, PI 473385 and PI 546023, these samples remained fully intact following the enzyme treatment and vortexing, indicating minimal digestion. It seems plausible that an alteration of the digestion solution, or addition of other enzymes commonly used in protoplast generation, may yield greater decomposition and thereby allow these genotypes to be evaluated as well. As previously mentioned, the diversity panel yielded greater variation between samples of the same genotype than either the cv. Superior tissue or the root crop panel experiments. We believe this was caused by a few separate factors: sampling, size, and quality. As these were wild and semi-domesticated genotypes, large, well-storing tubers were not consistently available for all accessions and the diminutive size of some of the tubers precluded sampling of specific tissues. Therefore, we elected to sample all tissues via a longitudinal core. This allowed us to maintain a consistent approach for tubers of all sizes but may have introduced greater variation if there were different relative abundances of tissues between tubers in a given genotype. As mentioned previously, the tubers exhibited various defects which may or may not have been present in all tubers of a genotype, perhaps contributing to sample variation as well.

### Cultivars and root crop species

The collection of grocery store tubers or roots of other starchy crops indicated the broad applicability of the protocol for potato cultivars with little regard of growing conditions or storage but limited suitability to other starchy crops. While sample culture conditions for this experiment are unknown and environmental factors such as stress are known to influence EI [27]; the significant differences observed between the pith of the various tuber accessions likely indicate that variation for EI present within cultivated germplasm allows the possibility of selection. However, whether selection for greater endoreduplication would actually yield plants that produce larger tubers or higher yields remains to be seen. What is apparent is that all of our potato tuber samples were highly endoreduplicated and a reliable protocol permits the study of genetic control of the process. While sweet potato and turnip reacted well, cassava, beet, and carrot yielded no usable results. This indicates that other, untested, starchy crops may or may not be responsive. The stark contrast between EI values in potato pith, where the lowest variety (red potato) was 1.56, and the other crops which ranged from 0.22 (sweet potato) to 0.87 (turnip) may be related in part to their differences in organogenesis; While potato tubers originate from stolons, a modified subterranean stem, the other organs are modified tuberous roots. While tuberous roots such as sweet potato may gradually swell over the lifecycle of the plant, bulking of potato tubers is a relatively rapid process, perhaps requiring a more acute manner of starch deposition and cellular expansion made possible by dramatic increase in nuclear DNA content. For instance, signs of sweet potato storage root initiation can be observed in as little as 13 days after transplant despite a growth period ranging from 90 to 150 days with an average as high as 140 days in some areas, not including growth that occurs prior to transplanting [28–30]. Conversely, potato tuber initiation is highly dependent on photoperiod and occurs later in plant development compared with sweet potato [31]. For instance, cv. Russet Burbank may take around 9 weeks after planting to initiate tuberization when grown in North America despite being a late maturity variety with a time requirement similar to that of sweet potato [32]. Evidence for a negative correlation between endoreduplication levels and duration of organ development has been presented in fleshy fruits and a similar association may be uncovered given more investigation of tubers and tuberous roots [33].

## Conclusions

 Here, we have demonstrated a novel procedure for evaluating the endoreduplication levels of tubers of potato, its close relatives, and some starchy root crops. The procedure, while time consuming, is reliable, relatively straightforward, and somewhat accommodating of common tuber defects. Nevertheless, it is possible further alterations may be made to increase throughput, minimize costs or further expand its application to other crop species. In establishing the efficacy of the protocol, we observed significant differences in endoreduplication between tuber tissues, cultivars, wild accession and species while there was no apparent difference between mature tubers of different sizes of the same variety. This protocol, in addition to the results we report, may serve as an early glimpse of the variation of endoreduplication in starchy root crops which may have significant agronomic relevance.

## Additional files

**Additional file 1.** A simplified overview of the entire tuber protoplast flow cytometry protocol. The detailed protocol is described within the text.

**Additional file 2.** The raw data of the tissue, diversity panel, and root crop experiments. It contains the relative abundances, in percentage, of each C-value peak as well as the EI value calculated from those percentages.

## Abbreviations

EI: endoreduplication index
PI: propidium iodide or plant introduction, depending on context
PIS: plasmolysis incubation solution
PW: plasmolysis wash solution (modified incubation solution)
AU: arbitrary units
C-value: (DNA content value)
