# Supplementary material for: Protoplast isolation prior to flow cytometry reveals clear patterns of endoreduplication in potato tubers, related species, and some starchy root crops
Source: Plant Methods. 2017 Apr 14;13:27. doi: 10.1186/s13007-017-0177-3 (PMC5391561; doi:10.1186/s13007-017-0177-3)
Supplement: Supplementary file 1 — Additional file 1. A simplified overview of the entire tuber protoplast flow cytometry protocol. The detailed protocol is described within the text. [file 13007_2017_177_MOESM1_ESM.pdf]

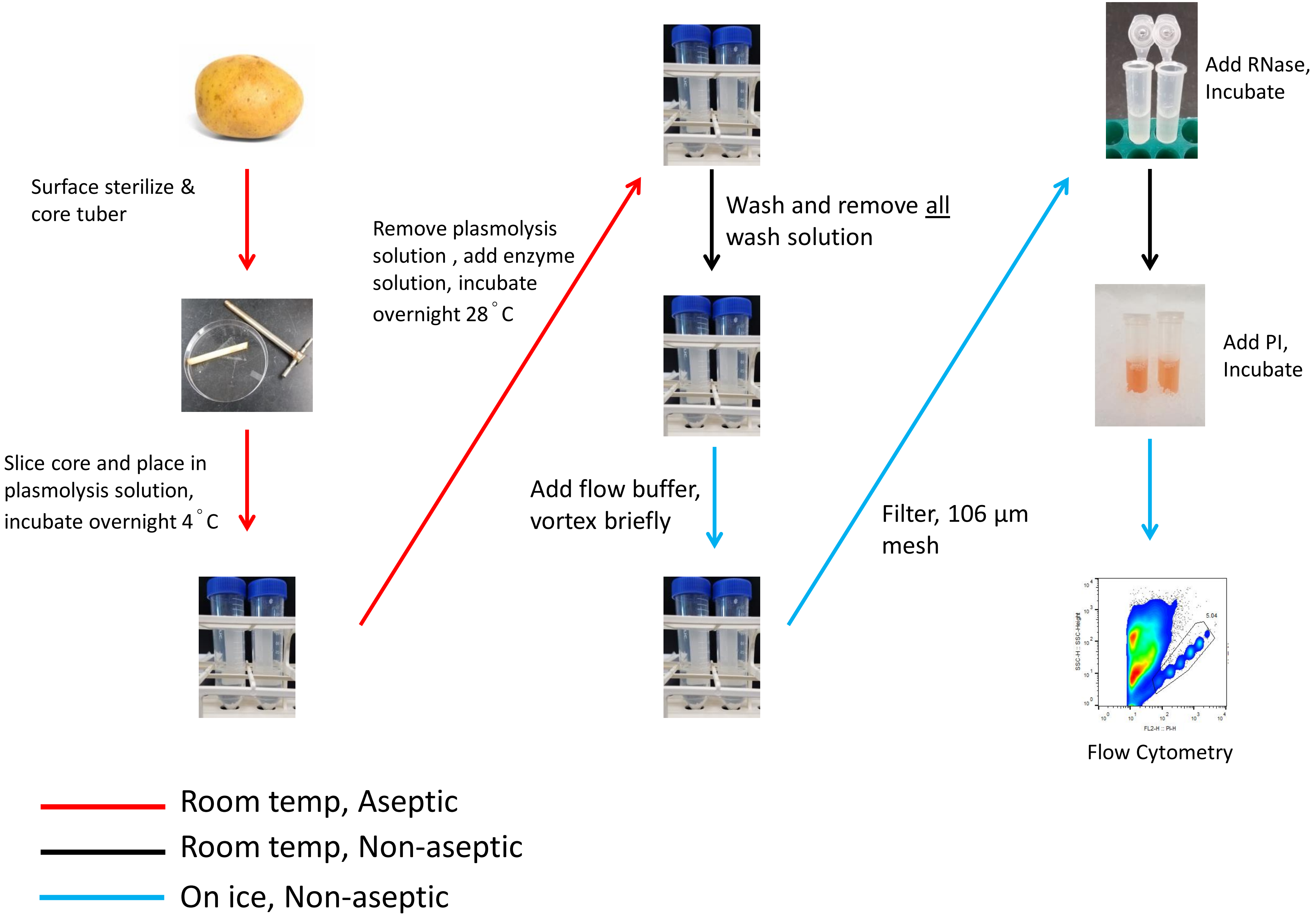
Surface sterilize & core tuber
Slice core and place in plasmolysis solution, incubate overnight 4 °C
Remove plasmolysis solution , add enzyme solution, incubate overnight 28 °C
Wash and remove all wash solution
Add flow buffer, vortex briefly
Filter, 106 µm mesh
Add RNase, Incubate
Add PI, Incubate
Flow Cytometry
Room temp, Aseptic
Room temp, Non-aseptic
On ice, Non-aseptic
